# Using science and psychology to improve the dissemination and evaluation of scientific work

**DOI:** 10.3389/fncom.2014.00082

**Published:** 2014-08-19

**Authors:** Brett T. Buttliere

**Affiliations:** Department of Methodology and Statistics, Tilburg UniversityTilburg, Noord-Brabant, Netherlands

**Keywords:** open science, publication process, scientific communication, choice design

## Abstract

Here I outline some of what science can tell us about the problems in psychological publishing and how to best address those problems. First, the motivation behind questionable research practices is examined (the desire to get ahead or, at least, not fall behind). Next, behavior modification strategies are discussed, pointing out that reward works better than punishment. Humans are utility seekers and the implementation of current change initiatives is hindered by high initial buy-in costs and insufficient expected utility. Open science tools interested in improving science should team up, to increase utility while lowering the cost and risk associated with engagement. The best way to realign individual and group motives will probably be to create one, centralized, easy to use, platform, with a profile, a feed of targeted science stories based upon previous system interaction, a sophisticated (public) discussion section, and impact metrics which use the associated data. These measures encourage high quality review and other prosocial activities while inhibiting self-serving behavior. Some advantages of centrally digitizing communications are outlined, including ways the data could be used to improve the peer review process. Most generally, it seems that decisions about change design and implementation should be theory and data driven.

Previous research has outlined the problems with the current publishing system and made suggestions about how to improve the system (Gottfredson, 1978; Rosenthal, 1979; Ioannidis, 2005, 2012a,b; Benos et al., 2007; Björk, 2007; Birukou et al., 2011; Simmons et al., 2011; Bekkers, 2012; Giner-Sorolla, 2012; John et al., 2012; Kriegeskorte et al., 2012; Lee, 2012; Nosek and Bar-Anan, 2012; Asendorpf et al., 2013). Instead of outlining this work, here I examine how the scientific literature (especially Psychology) can help us understand the problems and develop/implement more effective solutions.

## What is the real problem science faces?

The real problem for scientific communication and society more generally is the desire for success and power (or the desire to avoid failure) which prods human researchers to put their own interests above the interests of the group (Hardin, 1968; Skinner, 1972; Fehr and Fischbacher, 2003; Elliot, 2006; Thaler and Sunstein, 2008); the publishing system is only the obstacle this drive must overcome. The dilemma is that in order to advance, or at least keep, our careers, we must publish in high impact journals. There is competition to publish in these journals and people naturally began looking for a way to get the edge on the competition (Bentham and Mill, 2004). As those who bent the rules had better outcomes, the practices became normalized over generations, resulting in widespread “questionable research practices” (QRPs; Darwin, 1859; Skinner, 1972; John et al., 2012).

This motivation to get ahead is (probably) not a bad thing; it is what drives Science and human progress in the first place. The problem is an ineffective reward system which makes doing the prosocial action (e.g., no QRPs, open data, no file drawer, open methods) bad for the individual because it is less efficiently achieves high impact work and thus promotion. The goal here is to recast the system, the “game” the individual plays, such that working toward the individual success is also working toward the group’s success, or at least that individual success is not achieved at the expense of the group (Skinner and Hayes, 1976; Thaler and Sunstein, 2008).

## Designing successful change

There are many ways to institute behavioral change, but history and the psychological literature suggest that motivating change with reward is more effective than motivating change with punishment, which basically creates better cheaters and even encourages the behavior (e.g., prohibition, war on drugs, war on terror; Skinner, 1972; Nadelmann, 1989; Sherman, 1993; Higgins, 1997; Bijvank et al., 2009; Branson, 2012). Instead of focusing on creating tools to go back, catch, and thus punish (through reputation costs) previous scientific wrongdoers (Francis, 2012; Klein et al., 2014; Simonsohn et al., 2014), it would be better to focus forward on creating a system, incentive structure, and zeitgeist where the behavior is not continued (Gibbs et al., 2009); this is the goal below.

This is not a new goal, and many initiatives are attempting to stimulate prosocial behavior using rewards (Hartgerink, 2014). Unfortunately, without coordination, the effort to buy in quickly outweighs the expected utility, limiting engagement (Kahneman and Tversky, 1979). Many competitors divide the manpower and no tool has either all of the features that the scientist wants or the widespread acceptance which ensures it will be useful in the future. Initial step costs are also quite high, as for each new system the researcher must invest hours to set up their profile, learn the interface, and build up their network. These issues (e.g., high initial buy-in cost, divided utility/market, uncertainty of the payoff) help to explain why psychologists, despite verbally endorsing change, are not meaningfully engaging with current change initiatives (Buttliere and Wicherts, in preparation; Kahneman and Tversky, 1979). Research has demonstrated that too many options, especially for important choices like a retirement savings account, paradoxically leads to less participation (Iyengar et al., 2004).

In order to surmount these problems, open science tools should work together, putting aside individual interests and combining utilities in order to make the prize larger and lower the cost of achieving that prize. The most successful technologies are those that are so useful that people make time to learn and utilize the tool on their own (e.g., the printing press, the telephone, the internet, or Facebook, which is accessed more than 20 billion minutes per day; Deci, 1971; Skinner, 1972; Legris et al., 2003; Smith, 2014).

## A psychologically designed system

The goal here is to make a tool so useful that researchers make time to learn and utilize it on their own (like the microscope, the Likert scale, or QRPs; Legris et al., 2003). The tool should also endorse group centered behavior while inhibiting self-centered behavior (Skinner, 1972). While there is much discussion about the specifics of this ideal tool, it probably involves the internet and emulates the most successful social media technologies in utilizing: an attractive, easy to navigate, profile (e.g., osf.io, Academia.edu, Frontiersin.org, Facebook.com), a feed of targeted science stories based upon prior clicking behavior (e.g., RSS feeds, Facebook.com, Twitter.com, Frontiersin.org; Lee, 2012; Nentwich and König, 2014), a sophisticated rating/comment mechanism for content (e.g., Reddit.com, PLoSONE.org, F1000.com, PubPeer.com; Birukou et al., 2011; Hunter, 2012), and a new set of impact metrics which make use of the information available within the system (e.g., Klout.com, AltMetrics.org, Researchgate.net; Walther and Van den Bosch, 2012).

The basic reinforcements for the system are probably also the same as Facebook and Twitter, namely: the high quality, targeted, content provided in the newsfeed (Bian et al., 2009) and the good feelings we receive when notified that others have interacted with our content (Berne, 1964). These immediate reinforcements, paired with an easy to navigate user interface, are powerful enough to make Facebook users log in an average of 14 times per day and have researchers talking about Facebook addictions (Andreassen et al., 2012; Cheung et al., 2013; Taylor, 2013).

When an individual posts a paper, dataset, general comment, or new protocol to their profile, it shows up in the newsfeed of those the system believes will find utility in that content (e.g., collaborators, colleagues, researchers who click similar stories) and these people can view and publically comment on the work. When an individual interacts with a post, the system notifies the original poster (providing utility) and is more likely to display content from the same source again. The feed can also contain low key targeted notifications for professional organizations, conferences, special issues, and other services which notify the researcher of upcoming opportunities (again, utility) while also helping to pay for the system, potentially paying for the system outright.

Centralizing and digitizing the discussion of a post is probably the best part, as it provides the data upon which to generate the feed and saves readers much time otherwise spent thinking about things which have already been thought about (researcher’s rewarded for providing links and information which is “liked” by others). For instance, one could go to a paper or subfield and see if anyone has mentioned Cognitive Dissonance Theory, join the conversation, or start their own discussion with the authors/ community. While some may worry that reading this information adds extra work, protestations of data overload can be dealt with by first pointing out that we only need to “read it if we need it”, but also that the system will include sophisticated methods for discovering and readily presenting the highest quality content (e.g., Reddit, Facebook Lookback; Yarkoni, 2012).

When the researcher has a question they cannot find in the discussion of a paper or (sub)field, the system could suggest a list of experts who are likely to have the answer to that question (the expert is rewarded for answering these questions). This system could be keyword driven, pulling theoretical, methodological, and analytical keywords out of the researcher’s papers to create profiles (Pennebaker et al., 2001). These profiles can, in addition to matching experts with questions and improving the feed, speed along article/ researcher processing for meta(science) analyses and create network maps, similar to social media maps for summarizing literatures and fields more efficiently (Gilbert and Karahalios, 2009; Hansen et al., 2010).

## Good for the group

Information contained in the system can also be used to reward group based behaviors that are currently underperformed (e.g., making datasets/stimuli available, reanalyzing data, writing quality reviews). Impact metrics, instead of using only citations, can utilize all of the data in the system including: the impact of the individual’s work (e.g., shares, comments, ratings, who made those ratings), the impact of their comments on other’s work, whether data and syntax are uploaded, how well their interactions predict the general community’s, how they answer questions they are asked, and much more (Florian, 2012; Kriegeskorte, 2012).

The publicity of the comment section also means that the individual can develop a reputation and accrue an audience, driving impact. For instance, if one knows that certain researchers check the methods and statistics of new papers, replicates them, or just makes good comments, one may look for their comment when reading/citing a new paper (though the system itself could also have a built in statistics checker; Wicherts et al., 2012). The original author wants those researchers’ helpful comments and thus uploads the materials, data, and syntax for them to check (besides being rewarded directly for it). The methods checkers and replicators are motivated to do a good job as it is their reputation and the reader benefits enormously because they can trust that the effects are replicable and as reported (Yarkoni, 2012). Even if the reader doesn’t explicitly endorse the comment (e.g., like, sub comment), by searching for the author’s name in the comments or viewing the (statistical) replication page, reward can be administered. Because the individual can become impactful by engaging in these prosocial activities, the need for QRPs is alleviated while also making them harder to engage in, because people are rewarded for checking.

The system outlined above could be implemented without changing the fundamental peer review system. The proposed changes are expected to improve the system by encouraging, through quality impact metrics (Priem et al., 2010; Kreiman and Maunsell, 2011; Yarkoni, 2012), open practices and endorsing group centered behavior. Unfortunately, only adding this to the current system still looks backwards and does not deal with the competition to become published, the time papers spend waiting for reviews (Peters and Ceci, 1982), or the excess cost of the current system (Rennie, 2003; Edlin and Rubinfeld, 2004). It is time to examine how the data within this system could help improve the peer review mechanism.

## More impactful (read: important) changes

The changes suggested here are the most sensitive to small design flaws, which, over the decades, will grow as the current issues have. For this reason, it is imperative that we have a spirited debate about the specifics outlined below and not believe that our decisions are set in stone when we make them. Only continual maintenance of the system will ensure fidelity over time (Blanchard and Fabrycky, 1990).

Others have already outlined several alternative mechanisms by which to evaluate research including open review, review conducted by specialized services, and various levels of pre and post-publication review (Kravitz and Baker, 2011; Hunter, 2012; Kriegeskorte, 2012; Nosek and Bar-Anan, 2012). While it is still unclear how to keep bias out of the review services or reviews in general, we would like to suggest that the data within the current system can be utilized to facilitate review (Kreiman and Maunsell, 2011; Lee, 2012; Zimmermann et al., 2012).

When a researcher wants to publish a paper, the system could automatically send the paper to field experts, “rival” field experts, non-experts, methods experts, and statistical experts, based upon the data in the system (Kravitz and Baker, 2011). Reviewers can be asked to write brief reviews and make quantitative ratings of the paper or they can simply be presented with the paper and the system can see how they react (as ignoring the piece is also informative; Birukou et al., 2011; Kriegeskorte, 2012; Lee, 2012). These reviews can be done “pre-publication,” where reviewers privately provide feedback (while being rewarded through a counter on their profile), or the reviews could become immediately public and serve as the basis of discussion after a certain number of comments have been accrued (in order to avoid the anchoring effect of a bad first comment; Bachmann, 2011). If the paper is received well, it can be suggested to more individuals and groups that might also find utility in the paper.

Professional organizations maintain their role as disseminators of content (what they were originally designed to do; Benos et al., 2007), but would no longer be responsible for evaluating, reviewing, and publishing these works. Dissemination decisions can be made by editors, or the professional organization could use a computer and stipulate that in order for a paper to be considered for dissemination, it has to have certain keywords and have had × number of members comment on or like it, including some with higher impact factors. Each organization can have several “journals”, each with their own reputation (e.g., finding the most cutting edge work, only promoting the future classics, only promoting those that are preregistered). When a group promotes a work, the system sends it to those who are most likely to find utility in it, similar to the individual but on a much larger scale. The paper also earns a stamp of approval which grows (e.g., Bronze, Silver, Gold badges; Nosek and Bar-Anan, 2012) if the paper is received well and it is suggested to more users in the group; in this way the paper can “go viral”.

One further addition I would like to add to proposals that emphasize purely online review systems is the ability for, at the end of the year (or decade), extra badges to be given for the top 10 (or 100) papers published in a particular (sub)domain. These collections could be put together for any aspect of the paper (e.g., theory, methods, statistics), could be printed, and provide something to aim for in the creation of content besides high impact.

## Motivating change

Another aspect where Science can help is in getting people to adopt the system. Though open and post-publication review are popular among experts, a recent survey of 2,300 psychologists conducted by this author found that changes related to opening review were the three lowest rated potential changes to the publication system, with post publication review being rated 10th of 15 (Buttliere and Wicherts, in preparation). Change initiatives would benefit from empirically demonstrating the utility of the proposed changes, as has been done with opening review in the biomedical field (Godlee et al., 1998; Walsh et al., 2000; Pulverer, 2010).

We also know that scarcity increases the value of a good (Cialdini, 1993). When Facebook came out, it was only for Harvard students and for several years was invite only. Similarly, it may benefit a fledging open science platform to first be by invitation only, perhaps limiting access to those who supported the systems which combined to make it, and then only opening by invitation from those already in the system (like Facebook was). It should also be pointed out to field leaders and professors (who will get invited to the system earlier than others) that they serve as examples to others (especially students; Phillips, 1974) and that by not pursuing change for the better, they signal that nothing needs to be done and become a bad example (Darley and Latane, 1968). Obviously, marketing and advertising should also guide naming and implementation strategy.

Concerns about Science using behavioral engineering (Huxley, 1935; Rand, 1937; Orwell, 1949), are necessarily brushed aside by reminding ourselves that advertisers have been engineering us for their own profit since before Skinner outlined the methods in 1972. Behaviorally engineering a well-functioning system for ourselves would go a long way toward showing the public what the use of this technology for good looks like (Skinner, 1972; Thaler and Sunstein, 2008) and would very likely garner more trust and financial support in the future.

## In sum

There are many problems with the current academic publishing system, and many have suggested courses of action to solve those problems. Here I highlight science that can inform the discussion and decisions being made about these issues. Most importantly, humans are utility seekers and use whatever tools (e.g., QRPs) most efficiently help them achieve their goals. The reason psychologists are not engaging with change initiatives is because they have high initial step costs, and have uncertain outcomes due to a fragmentation of the market. I propose that open science tools put individual interests’ aside and work together to raise the utility and lower the cost of using the common tool. I next examined how the data from one, centralized, online system can be used to improve scientific communication by being immediately rewarding to the individual while also encouraging group-centered behavior and concurrently inhibiting self-centered behavior. There is much more conversation to be had, but I hope this essay will help focus conversation on using science to guide decision making.

## Conflict of interest statement

The author declares that the research was conducted in the absence of any commercial or financial relationships that could be construed as a potential conflict of interest.
