# Corrigendum: Using Science and Psychology to improve the dissemination and evaluation of scientific work

**DOI:** 10.3389/fncom.2015.00053

**Published:** 2015-05-08

**Authors:** Brett Buttliere

**Affiliations:** Net-based Knowledge Communication in Groups, Knowledge Media Research CenterTübingen, Germany

**Keywords:** science, open science, open data, using science to improve science, behavioral economics, psychology

The citation of Hartgerink (2014)

“Open science protocol,” in Poster Presented at the Annual Meeting of the Society for Personality and Social Psychology (San Antonio, Texas, US)

should instead be a citation to Bik and Goldstein (2013)

Bik, H. M., and Goldstein, M. C. (2013). An introduction to social media for scientists. PLoS Biol. 11(4), e1001535. doi: 10.1371/journal.pbio.1001535

## Conflict of interest statement

The author declares that the research was conducted in the absence of any commercial or financial relationships that could be construed as a potential conflict of interest.

